# Commission of Lunacy on Miss Amelia Maria Hortensia Collings

**Published:** 1848-07-01

**Authors:** 


### Medical Jurisprudence in relation to Insanity.

### COMMISSION OF LUNACY ON MISS AMELIA MARIA HORTENSIA COLLINGS,

On Friday, the 4th of February, 1848, at the Roebuck Tavern, Chiswick,

*Before* Francis Barlow, Esq., *Master in Lunacy, and a Special Jury.*

Mr. Temple, counsel, and Mr. W. H. Rymer, of Chancery-lane, solicitor, appeared in support of the Commission; and Mr. Miller, counsel, and Mr. Moore, solicitor, on behalf of Miss Collings. The Master briefly opened the Commission, and Mr. Temple addressed the jury, referring principally to the points sworn to by the witnesses.

### MEDICAL EVIDENCE.

Dr. Hodgkin examined.—Had been in the habit of attending cases of lunacy. Had seen Miss Collings frequently. He saw her by the order of a gentleman named Carrè, at the asylum in Tokenhouse-yard. He spoke of her as an alleged lunatic, and spoke of her sister at the same time. He does not consider her a person to be trusted with the government of herself and property. Saw letters that she had written. Was shown some letters to Lord John Russell and Mr. Goodman; the letters were directed to the Mansion House. The letters show the delusions under which she labours. In the letters, she and her sister complained greatly of being persecuted. They thought they were descendants of the royal house of Spain, and entitled to large property in Guernsey. She very distinctly averred that Louis Philippe was going to take possession of the island of Guernsey, and that her mother had been murdered. She said she was a descendant of the Bourbon family. She had a delusion that the life of a person named Dobree was in jeopardy, and that he was, in fact, to be assassinated, and she desired me to warn him that such would be the case. She implied that I should be assassinated myself if I did not obey her commands. (Laughter.) She is clearly not in a rational state of mind, and therefore I do not think it would be proper to set her at liberty. On two occasions I received pretty strong proofs of her delusions, for she struck me twice with her fist. (Laughter.)

Dr. Winslow, of Sussex House, Hammersmith, examined.

Mr. Temple.—When did you first see this young lady ?

Witness.—I first saw her on the 28th December last, at the Asylum.

Mr. Temple.—Well, and what did you think then ?

Witness.—She appeared quite incapable of exercising any continuity of thought, or sustaining a connected or rational conversation. She said she was entitled to large property; and upon being asked why she thought so, she said that it was owed her by the traders of Guernsey ; that it was not only owed to her, but also to her sister. She said she had been entrapped into a lunatic asylum ; that Don Carlos had a great deal to do with her affairs ; that her mother had been cruelly murdered by a smuggler, in the Island of Guernsey, to get possession of her property. She said that her sister, Maria, had been placed there, and had been sold to the proprietor of the asylum for 1500*l.*, and that the whole of her affairs were known to Governor Napier and his wife ; and that if he did not release her from confinement, there were parties who would run him through the body. Again, on referring to the murder of her mother, she said that the whole island were well acquainted with the fact. She also represented that she was under the special protection of parliament, and that she had been poisoned by the "*arsenite of copper.*" She said it had been infused into her food. She then observed, that her mother was "*Her Majesty of Spain,*" and that her sister could at any moment be the "Princess of Spain;" and she stated that ten millions of money had been spent in the traffic of the slave trade ; she stated that Louis Philippe had sunk "The Avenger," with three hundred passengers on board. I consider her certainly labouring under delusions, and of unsound mind.

Mrs. Euphemia Findlater, the sister, was then called, and examined at some length.—Her evidence strongly substantiated the existence of positive delusions of mind, and incompetency of her sister to manage herself and her property.

Mr. J. Bolling, surgeon, of Hammersmith, examined.—Had been in the habit of attending to cases of lunacy for twenty-eight years. He saw Miss Collings on the 8th of December last, in company with Dr. Winslow. Had seen her a second time. Had examined her state of mind, and considered her of unsound mind, and incapable of taking care of herself or her affairs. She declared that she was worth 100,000*l.* The topics of the conversation were the imaginary conspiracy of Collings to rob her of her property ; and that Joseph Collings was agent of Louis Philippe, but that he was too deep for Louis Philippe, inasmuch as he had got the greater portion of the property for himself. These were the delusions under which she laboured. That her mother had been murdered, that every one in Guernsey knew of the event that her mother had been murdered, and that the principal actor in the affair was a person named Mansell. She accused all parties in Guernsey with conspiracy, and in having instigated the act. With regard to money, I put this question to her: "Was I right in understanding you, that you considered yourself entitled to 10,000*l.*?" And she said, "Instead of it being 10,000*l.*, it is 100,000*l.*" She said, "It's more than that —it is a million ;" and if the Chancellor was not satisfied with the information I had had, he was to apply to the premier, Lord John Russell, who would give him further information.

### EXAMINATION OF THE LUNATIC AT THE ASYLUM.

Mr. Temple.—If anybody has any questions to put to this young lady when she comes, it will be put through you, Mr. Commissioner.

The Learned Commissioner.—Oh, yes, that is quite right, and I think it will be the best way to manage.

Mr. Temple.—Yes ; I think the questions on business had better be conducted *secundem artem.*

Miss Amelia Maria Hortensia Collings was then introduced ; and after she had seated herself, gazed round the room, looking at the jury, and appeared somewhat excited, when

The Commissioner said—We have come here to-day by the authority of the Chancellor, to inquire into the state of your mind, to know whether you are capable of taking care of yourself, and therefore you will excuse me in asking you some questions. The Chancellor has authorized these gentlemen to come. We are told that you consider yourself entitled to a very considerable property.

Miss Amelia Collings, looking towards Mr. Miller, said—I believe that gentleman will answer the questions.

But we prefer the lady's opinion. We would rather have your answer, if you please. The Chancellor himself wishes to know, too.—Oh, very well.

You cannot tell the amount of your property, can you? (A pause.) Has Sir William Collings anything to do with you?

Miss Collings (hesitating)—

Was he not your guardian?—He was in former years; but he was not—so—(hesitating)—beyond a certain age.

Did he assign any reason for that?—That's sufficient; the matter will be taken up by and by.

Did he give you any account of your property?—He acted like a fool!

Then you ought to have acted otherwise. What do you think the amount of it is?

Miss Collings (looking vacantly around her) ejaculated, in a somewhat wandering manner—It was an infamous theft.

Commissioner.—He acted you say——

Miss Collings.—(Quickly.) It was an infamous theft.

Commissioner.—Who can give us any account of your property?—Upon another occasion you will receive more.

Very well, then. They deal with you here with very great kindness, don't they?—Would be all very well if there was not corruption and bribe in the way. If Sir William Collings had been out of the house, it would also have been very well.

Has he agents here?—Yes, yes; of course he has agents.

But Sir William Collings is in Guernsey. Did Mr. Dobree come here to see him?—Mr. Castcar, a linen-draper in London, came here and called himself Dr. Hodgkin.

Do you see Sir William Collings here?—Dr. Winslow is the only medical man that comes here at all.

Are you quite sure of that? Are you quite sure this man calls himself Dr. Hodgkin?—(Excited.) I having nothing to say to you. I have seen him at Mr. Dobree's, the merchant, as his clerk.

Some of those letters which they have shown to us, we are told, were written by you. Is it so?

Miss Collings paused.

Commissioner.—They are signed "Amelia De Bourbon."—"Miss Amelia," the writers of the letters——They are not opened at present; but they are written——

Do you mean we cannot understand them?—The letters have not been received by the persons to whom they were addressed. I have one directed to Mrs. Findlater at Montpelier.

Do you remember writing to her?—Oh, yes; she ought not to have exposed it. It was very injudicious of her, and at the same time very indiscreet, to show her letters to anybody save the lady to whom it was addressed.

The Commissioner showed her a letter, and she replied—

It is my own letter. She must have been mad to produce it.

Commissioner.—But could you explain what it means? Some of the letters are about a conspiracy against you.—Miss C——. It's all Sir William Collings from the beginning to the end, and, as the Lord Mayor calls him, the "*Bilk*," and very properly so, because he is a very fraudulent person. Ask the Lord Mayor.

Now, about your mother?—I should think that the letter has an account, probably, of the loss of my mother.

Mr. Temple.—No, I believe not.

Miss Collings.—I believe it has; I believe the subject of this letter has something about my mother's house. My mother died in Guernsey in '29.

Commissioner.—Well, did——

Miss Collings (interrupting.)—Yes, yes, to be sure.

Commissioner.—What makes you think that she died at Guernsey?—Why, what do you ask me that for? Is it an important question to ask?

We are told you think Sir William Collings had something to do with her death?

We hear you think that he was the instigator of her death? How did he instigate her death?—Eh? what?

How did he instigate her death?—He *did* instigate her death, and *that is certain. He did, that's certain.*

And when did it take place?—Why?

It took place in India, did it not?—You appear to have seen Sir William Collings. Of course you have been long employed by Sir William Collings, and therefore that makes you say so.

I assure you I never saw Sir William Collings. There is a paper.——That is a forgery—a forgery—a forgery.

I give you the reason why I think she died at Madras, in India.—My mother was dead in that year. That deed has been lately made.

What makes you think she died at Guernsey?—Well, the truth is, that the country people there told me of it.

Who told you so?—(Excited.) They all told me so.

But didn't your sister, Mrs. Findlater—(interrupted.)—The country people all told me so, and therefore she is a *liar* in this case. I believe she was murdered.

Did your mother make any will?—My mother's will was of no great effect.

Did she have any property?—Before she was murdered—and there was a very large company together the night when she was murdered.

Have you not something to do with the will?—Yes, I have—that is all Sir William Collings's business.

Do you recollect having seen him?—As the Lord is my judge.

The doctor says you think that you have been poisoned. Is it so?—I believe something like a letter was written upon the subject of poisoning.

Do you think you have been poisoned since you have been in this house?—(Emphatically.) Yes, I do.

Had you any impression that you had any poison put in your food?—I have nothing to do with that.

Dr. Hodgkin says you told him that you had arsenic put in your food?—You must ask some one else.

Have you any connexion with Louis Philippe?—Probably you may have that honour. (A laugh.)

No, I have not, I assure you.—(Smiling.) Well, ask Lord John Russell that question.

Will he tell us that?—(Angrily.) I cannot answer I am not answering to that. I cannot say——

If I could, I would ascertain what your property consisted of. Have you any account of it? Do you remember being at Rome?—I went, of course, to my sister.

We heard you had been there.—Well, that is quite sufficient now.

Have you heard any account of the destruction of the AVENGER, in the Mediterranean, Miss Collings? (A pause.) Do you remember reading an account of the destruction of the AVENGER in the Mediterranean?

Miss Collings was silent.

Had Louis Philippe anything to do with it?—Certainly; evil people had to do with it.

Had Louis Philippe anything to do with it?—I am no accuser of the people.

Did you not tell somebody of it?—I have never said it.

Did you never say anything about it?—I am no judge, nor am I an accuser.

No, truly; but perhaps you may have said so accidentally. Have you not said that some one in the place knows all about your property?—Mr. Samuel Dobree was perhaps formerly a policeman; do you wish to see him?

What makes you think you are connected with the royal family of Spain?—Am I not my mother's own? (Laughter.)

Can you explain what connexion there was between you?—If your mother was Princess—that's quite sufficient, I should think? (A laugh.)

Do you think so? Have you reason to suppose that your mother was a Princess? —She was, I know—she *was* a Princess. She was a king's daughter—she was more than a Princess. She was grand-daughter of Charles, King of Spain.

You have no doubt about that?—No doubt about that.

How can you prove that?—Yes—yes.

Tell us what amount your property is.—What do you say now? What is it—what is it?

Is your property more than 100,000*l*., or what is it?—(Looking to her counsel.) Did you not, Mr. Miller, say it was 5000*l*., sir?

After a very brief deliberation, the foreman of the jury said—"We are unanimously of opinion that Amelia Maria Hortensia Collings is now of unsound mind, and incompetent to take care of herself and property, and that she has been so since the 4th of September, 1847."

---

